# Design of a Drop-in EBI Sensor Probe for Abnormal Tissue Detection in Minimally Invasive Surgery

**DOI:** 10.2478/joeb-2020-0013

**Published:** 2020-12-31

**Authors:** Guanming Zhu, Liang Zhou, Shilong Wang, Pengjie Lin, Jing Guo, Shuting Cai, Xiaoming Xiong, Xiaobing Jiang, Zhuoqi Cheng

**Affiliations:** 1 School of Automation, Guangdong University of Technology; 2 Department of Neurosurgery/Neuro-oncology, Sun Yat-sen University Cancer Center, State Key Laboratory of Oncology in South China, Collaborative Innovation Center for Cancer Medicine; 3 The Mærsk Mc-Kinney Møller Institute, University of Southern Denmark

**Keywords:** Laparoscopic surgery, drop-in sensor probe, electrical bioimpedance spectroscopy, discriminant analysis

## Abstract

It is a common challenge for the surgeon to detect pathological tissues and determine the resection margin during a minimally invasive surgery. In this study, we present a drop-in sensor probe based on the electrical bioimpedance spectroscopic technology, which can be grasped by a laparoscopic forceps and controlled by the surgeon to inspect suspicious tissue area conveniently. The probe is designed with an optimized electrode and a suitable shape specifically for Minimally Invasive Surgery (MIS). Subsequently, a series of *ex vivo* experiments are carried out with porcine liver tissue for feasibility validation. During the experiments, impedance measured at frequencies from 1 kHz to 2 MHz are collected on both normal tissues and water soaked tissue. In addition, classifiers based on discriminant analysis are developed. The result of the experiment indicate that the sensor probe can be used to measure the impedance of the tissue easily and the developed tissue classifier achieved accuracy of 80% and 100% respectively.

## Introduction

Laparoscopic surgery, also known as Minimally Invasive Surgery (MIS), is a specialized technique for performing surgery. During a laparoscopic surgery, a rigid viewing endoscope is inserted via a small incision adjacent to the umbilicus, and one or more accessory punctures are used to introduce various treatment tools for grasping, cutting, suturing and achieving hemostatic control. Compared to conventional open surgery, laparoscopic surgery can provide tremendous benefits to the patients such as less bleeding, smaller amounts of anesthesia, less pain and minimal scarring. These advantages make laparoscopic surgery a commonly selected method for surgical treatments inside the abdominal cavity. Particularly, laparoscopic surgery is often used for pathological tissue resection [1].

During a cancer surgery, making a close margin in the pathological area is critical, however, remains a big challenge because of complex pathological anatomy and tissue deformations [2]. If the cancer is not removed completely, the residual cancer tissue inside the patient's body may continue growing after the surgery, thus re-operation for removing the cancer tissue is often required. Unlike in an open surgery where the surgeon can palpate and feel the tissue, the surgeon operating a laparoscopic surgery can merely rely on the vision from the endoscope to identify the pathological tissues. Conventionally, a pathologist examines the removed tissue (during or at the end of the surgery) to be sure that all cancer cells have been removed. However, the pathological examination of the resection margin will slow down the entire surgical procedure significantly [3]. Therefore, the surgeon is often required to cut out a big rim area of the healthy tissue around the pathological area to ensure the cancer is completely removed.

To address this challenge, advanced sensing technologies have been developed for tissue detection, aiming to make the surgery safer and more effective. One of the well-known commercialized examples is the Firefly Fluorescence Imaging system (Intuitive Surgical, Inc., U.S.), which use near infrared illumination to enhance the imaging contrast between the pathological tissue and the normal tissue which are dyed by fluorescent agent. In addition, BK Medical Inc. develops a drop-in ultrasound transducer (X12C4, BK Medical Holding Company, Inc., U.S.) which can be grasped by a robotic forceps and controlled by the surgeon to examine the area of interest. The effectiveness of both technologies was demonstrated [4,5]. Apart from the Fluorescence imaging and ultrasound transducer, some tactile and force sensors have also been designed to provide the touch feel during the operation, thus the surgeon may palpate the tumor based on the stiffness difference between the cancer tissues and the normal tissues [6,7,8]. Based on the designed tactile or force sensing, considerable research efforts have been made for tumor localization in MIS, such as in [9] a haptic palpation probe is designed to locate the subcutaneous blood vessel in robotic assisted MIS; in [10] a novel robotic sweeping palpation method is proposed for digital rectal examination (DRE); and in [11], force sensing uncertainties during palpation is considered and a compensation framework is proposed to achieve accurate palpation. However, the above-mentioned devices or designs either are too expensive or specifically be used for robotic surgery; few of the sensor probes are available for normal laparoscopic surgery.

Recently, Electrical Bio-Impedance (EBI) sensing technology has been noticed to provide extra ability to identify different tissue types based on the tissue's electrical property which the human cannot naturally perceive [12,13,14,15]. It has been reported that the EBI sensing can be used to detect the cancerous tissue in varies of organs areas effectively such as kidney [16], oral [17], neck [18], liver [19], brain [20] and skin [21]. Furthermore, the advantages such as low cost, easily miniaturization, non-invasive or minimally invasive, and real-time detection of this sensing technology have attracted ample research attention.

Although several EBI sensing devices have been proposed and developed in previous studies [22,23], they are generally large. As for some other existing electrode probes such as ZedScan (Zilico Ltd.) and in [36, 37], the design of the electrodes may not fit to the MIS environment. Specifically, the electrode in previous studies were designed as 2-needle electrodes [24], concentric needle electrode [25], and 2 ball electrodes [26]. In study [27], the EBI sensing was proposed to be integrated directly onto the bipolar forceps jaws. However, these electrodes configurations commonly have concentrated sensitivity about a point. One possible way to improve the signal-noise ratio of the measurement can be done through distributing the sensing electric field in an area. In addition, the objective of this study is to develop a sensor probe based on the EBI sensing technology with considerable minimal size and specifically designed electrodes. The probe can be grasped by an endoscopic forceps and placed on the surface of the target tissue to inspect the pathological tissue area for in site tissue information in real time.

## Sensor probe design

### Design requirements

**Fig.1:**
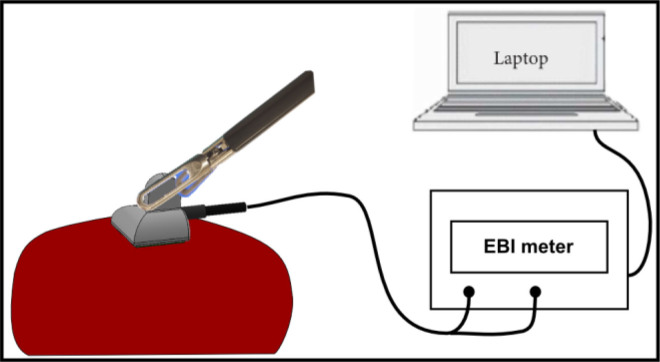
The proposed sensor probe aims to assist in the MIS for tissue detection. The probe connects to an EBI meter for measurement and the sensing data is streaming to a laptop for processing and display.

As shown in Fig. 1, the proposed drop-in probe aims to assist the surgeon in detecting abnormal tissue areas during a laparoscopic surgery. The surgeon can grasp it with a laparoscopic forceps and slightly press on the surface of the suspicious tissue area for examination. An EBI meter is connected to the probe for providing excitation signals and measuring corresponding EBI values. The measured EBI values are then sent to the connected laptop for data processing. With the constructed training dataset, a tissue identification algorithm is developed to distinguish the normal and abnormal tissue.

In order to satisfy the objectives mentioned above, the following criteria are considered in the design phase as listed below:

1. The size of the designed probe should be smaller than 12 mm so that it can enter the abdominal cavity through a trocar;
2. The EBI measurement should be conducted in multiple frequencies in order to accurately reflect the tissue's electrical property;
3. The probe should be made in a low cost and be able to merge to the current surgical environment easily.

### Modelling of tissue electrical bioimpedance

**Fig.2:**
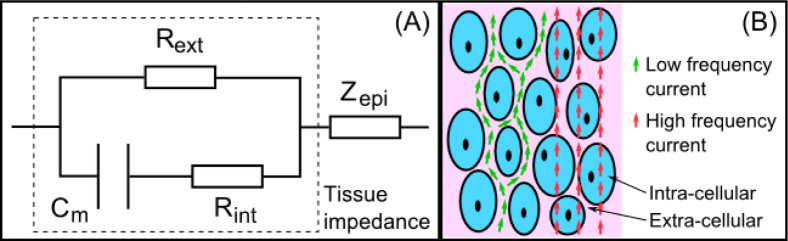
(A) The model of the electrical bioimpedance measured by the proposed sensor probe; (B) Modelling the low frequency and high frequency current passing through biological tissue.

The electrical bioimpedance of the measured value can be modelled as shown in Fig. 2(A). The overall electrical impedance value consists of the tissue impedance and the electrode polarization impedance *Z_epi_*. The tissue impedance is commonly described using the Cole model [28]. This model imitates biological structure constructed by a resistor *R_int_* simulating the intra-cellular component, a resistor *R_ext_* simulating the extra-cellular component and a capacitor *C_m_* simulating the cell membrane as shown in Fig. 2(A). In order to extrapolate the tissue's electric property, signals of multi-frequencies in the range from 1 kHz to 2 MHz, known as the *β* dispersion, are used for excitation. Within this range, high frequency signal is more capable to pass through the cell membrane and the measured impedance value involves more portions of the intra-cellular resistance, while low frequency current mainly passes the extra-cellular region and reflects the electrical impedance of this region.

In addition, the electrode polarization effect can influence the measured value significantly. The electrode polarization impedance is generated due to the double layer phenomenon in the contacting layer between the electrode and the tissue, which is known as the Gouy-Chapman effect [29]. The value of *Z_epi_* is found to be affected by several factors such as temperature, electrode materials, electrode geometry, tissue types, environmental pH, applied pressure, excitation waveform and excitation frequencies [30]. Thus, the electrode polarization effect is commonly considered as a main reason of variate in the measured EBI value.

### Electrode design

**Fig.3:**
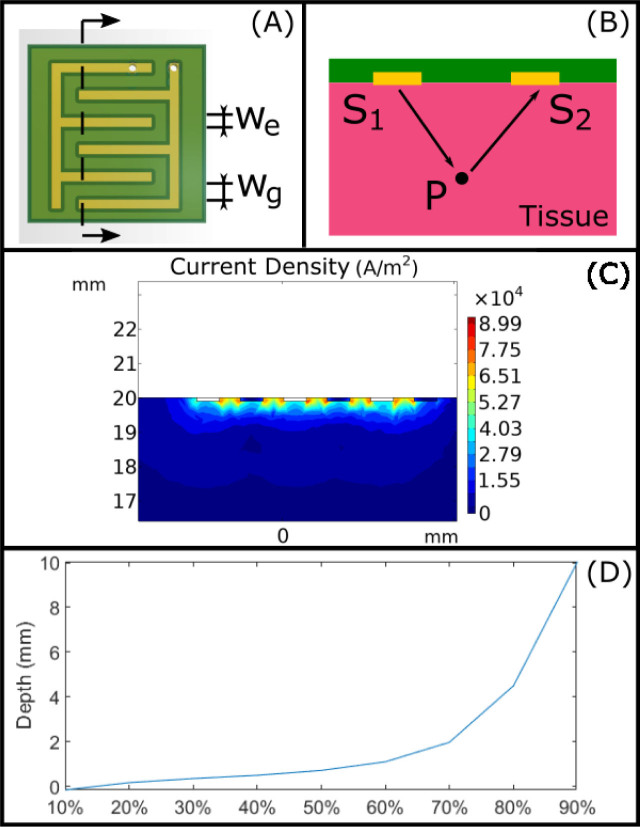
(A) The electrode pattern; (B) Modelling the EBI sensing of soft tissue with the designed electrode; (C) the current density simulation of the section indicated in (A).

Cross-finger electrodes configuration is often exploited for EBI sensing [15]. In a two-electrodes configuration, the excitation current is injected through the electrode pair, and the reciprocal current is measured through the same electrodes. Although the measured signal mixes both biological impedance and the electrode impedance when bipolar electrode configuration is used, this configuration enables compact design and simple circuit connection to an LCR meter. In this study, the electrodes are designed as a plate with a popularly used pattern of bipolar electrode as shown in Fig. 3. With this electrode configuration, the injected excitation current can be distributed evenly on the target tissue area. The size of the electrode is designed to be 7×9 mm, allowing it to enter the trocar and to facilitate the measurement on a relatively big area with good contact. It is necessary to mention that the key parameters of this pattern consists of the number of the fingers, and the electrode's width *w_e_* to the gap width *w_g_*. According to the study of Stulik *et al.* [31], having more fingers on the electrodes can not only increase the signal value but also the background noise, thus leading to no changes in signal-noise ratio. Meanwhile, wider electrode fingers provide more electrode surface area, but lose some of the radial diffusion pattern. Therefore, while the overall signal increases, nonspecific surface reactions (background signals) increase with a slightly faster rate, and further decreasing the signal-noise ratio. With fully consideration of the above-mentioned analysis, the electrodes plate is made with three fingers, the electrode width *w_e_* is set to be 24 mil and the gap width *w_g_* is set to be 48 mil.

The measurement sensitivity using the designed electrode is analyzed as follows. We assume that the tissue material is homogeneous. According to previous studies [32], the impedance of tissue is calculated as an integration of the sensitivity density distribution *S*, the tissue's electrical conductivity *σ* and the tissue's relative permittivity *ɛ* over the measured tissue volume *Ω*:

(1) $$Z=\int_{\Omega }\frac{1}{\sigma -j\omega \varepsilon }\mathrm{Sd}\Omega$$

where the sensitivity *S* for a bipolar configuration is equal to the square of the current density $J:S={\left|\vec{J}\right|}^{2}$ .

Furthermore, the inverse problem based on the designed electrode pattern is analyzed as follows. The inverse problem in the EBI measurement is the procedure to derive the material's impedance given the injected current value and the measured voltage. Here, we simplify the electrode pattern as a dipole model, in which the electric current flows into the tissue via the electrode pair. Assuming that the tissue does not generate induced current flow, the current density *J* is approximated to be proportional to the electric field strength *E*:

(2) $$\vec{J}=\left(\sigma -j\omega \varepsilon_{0}\varepsilon_{r}\right)\cdot \vec{E}$$

As shown in Fig. 3(B), the electric field of a random point *P* in the region caused by applied excitation current, can be calculated according to Coulomb's law as

(3) $$\vec{E_{p}}=\int_{S_{1}}\frac{dq_{1}}{4\pi \left(\sigma -j\omega \varepsilon \right)r_{1}^{2}}+\int_{S_{2}}\frac{dq_{2}}{4\pi \left(\sigma -j\omega \varepsilon \right)r_{2}^{2}}$$

where *S*_1_ and *S*_2_ are the area of two electrodes, respectively. *r_i_* (*i* = 1,2) represents the distance from an element on electrode *i* to point *P*, and *dq_i_* is the charge on this element.

Considering the complexity of the inverse problem, the Finite Element Method is utilized for the simulation based on COMSOL Multiphysics^®^ software. Through the simulation results of the current density distribution, the depth of the tissue to which the excitation current flows, can be estimated. The model is simplified by using direct current in the simulation. As shown in Fig. 3(C), the electrode pattern is placed on the top surface of the material, and a direct current is injected between the electrodes. The simulation results indicate that 80% of the energy is contained within the depth of 4.5 mm.

### Probe design and fabrication

**Fig.4:**
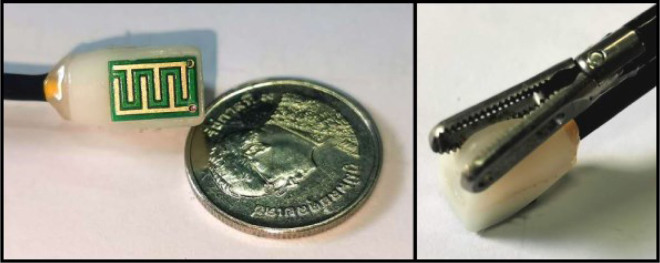
The prototype of the sensor probe, which can be grasped by a surgical forceps for measuring the area of interest.

A prototype is fabricated as shown in Fig.4, which is made as a piece of PCB, which potentially can be bio-compatible [35]. Gold plated electrodes are used in order to minimize the electrode polarization effect [38]. The casing is made by 3D printing due to the low-cost consideration.

A small extrusion structure is added to the casing to allow the force grasping it in different angles and manipulation. The width, the length and the height of the probe are 8 mm, 11 mm, and 16 mm, respectively, allowing it to pass through the trocar used for MIS to insert surgical tools such as laparoscopy. In addition, all the corners of the probe are rounded so as not to scratch the tissue during the manipulation.

### Algorithm for tissue classification

In order to judge the measurement for distinguishing between healthy tissue and pathological tissue, supervised machine learning algorithm is used. Then new sensing data can be estimated from the training dataset.

Discrimination Analysis is a supervised algorithm using information of classes to find new features in order to maximize its separability. Based on different feature assumptions, this classification method includes 2 main applied methods: Linear Discriminant Analysis (LDA) and Quadratic Discriminant Analysis (QDA). LDA assumes the feature covariance matrices of both classes are the same, which results in a linear decision boundary. In contrast, QDA is less strict and allows different feature covariance matrices for different classes, which leads to a quadratic decision boundary.

Both LDA and QDA works under the assumption that the data are normally distributed. Given an input *x*_0_, the response as in a Bayes classifier is predicted:

(4) $${\hat{y}}_{0}={\mathrm{argmax}}_{y}\hat{P}(Y=y|X=x_{0})$$

For a binary group classification, a data set can be defined as ${\left(x_{i},y_{i}\right)}_{i}^{n}$ where *x_i_* ∈ ℝ^*d*^ is the input, *y_i_* is the output, and *n* is the number of the data points. In this case, the input *x_i_* is a vector of measurements of different excitation frequencies [*R*_1_, *C*_1_, *R*_2_, *C*_2_, ... , *R_d_*_/2_, *C_d_*_/2_], of which *R* is the resistance and *C* is the capacitance. The output *y_i_* has 2 values, 0 and 1, representing normal tissue and pathological tissue respectively.

Here, we use the Bayes rule to obtain the estimate:

(5) $$\hat{P}(Y=k|X=x)=\frac{\hat{P}(X=x|Y=k)\hat{P}(Y=k)}{\hat{P}(X=x)}$$

The distribution of the EBI values in either group is considered as a Multivariate Normal Distribution: $\hat{P}(X=x|Y=k)={\hat{f}}_{k}(x)$ . In this case, the density function for class *k* satisfies

(6) $$f_{k}(x)=\frac{1}{{\left(2\pi \right)}^{p/2}{\left|\Sigma_{k}\right|}^{1/2}}e^{-\frac{1}{2}{\left(x-\mu_{k}\right)}^{T}\Sigma_{k}^{-1}(x-\mu_{k})}$$

where *μ_k_* and *Σ_k_* is the mean and the covariance matrix of the input for class *k*. In addition, we assume the initial degree of beliefs in either classes are equal, and thus $\hat{P}(Y=0)=\hat{P}(Y=1)=\Pi_{k}$ . Furthermore, Eq. (5) can be also written as:

(7) $$P(Y=k|X=x)=\frac{f_{k}(x)\Pi_{k}}{P(X=x)}=C\Pi_{k}|\Sigma_{k}{|}^{-1/2}e^{-\frac{1}{2}{\left(x-\mu_{k}\right)}^{T}\Sigma^{-1}(x-\mu_{k})}$$

where *C* = ((2*π*)^*p*/2^*P*(*X* = *x*))^−1^ is constant and keeps the same for both classes. Furthermore, we take the logarithm of both sides and we have:

(8) $$\log P(Y=k|X=x)=\log C+\log\Pi_{k}-\frac{1}{2}\log|\Sigma_{k}|-\frac{1}{2}\mu_{k}^{T}\Sigma_{k}^{-1}\mu_{k}+x^{T}\Sigma_{k}^{-1}\mu_{k}-\frac{1}{2}x^{T}\Sigma_{k}^{-1}x$$

In QDA, since the covariance matrix for both classes are different, the classifier *ℱ_QDA_* can be obtained as:

(9) $${ℱ}_{\mathrm{QDA}}=\log\Pi_{k}-\frac{1}{2}\log\left|\Sigma_{k}\right|-\frac{1}{2}\mu_{k}^{T}\Sigma_{k}^{-1}\mu_{k}+x^{T}\Sigma_{k}^{-1}\mu_{k}-\frac{1}{2}x^{T}\Sigma_{k}^{-1}x$$

While the covariance matrices of both classes are the same for LDA, namely *Σ*_1_ = *Σ*_2_, the classifier *ℱ_LDA_* can be simplified as:

(10) $${ℱ}_{\mathrm{LDA}}=\log\Pi_{k}-\frac{1}{2}\mu_{k}^{T}\Sigma_{k}^{-1}\mu_{k}+x^{T}\Sigma_{k}^{-1}\mu_{k}$$

## Experiment evaluation

### Experimental setup

**Fig.5:**
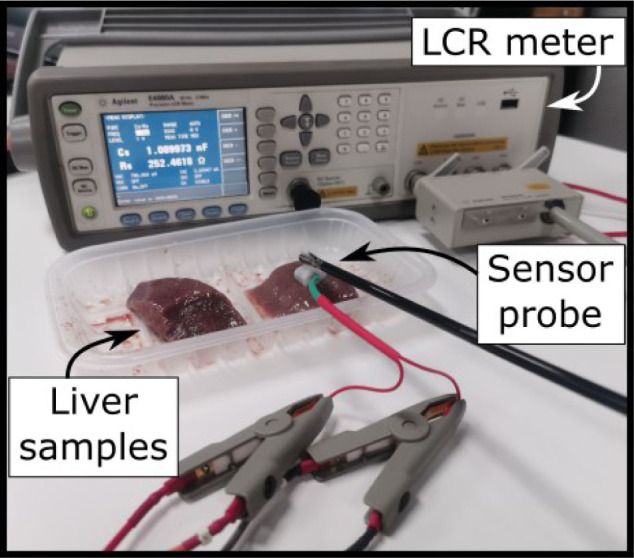
The experimental setup including an LCR meter, tissue samples and the designed sensor probe which is grasped by a surgical forceps.

The experimental setup is shown in Fig. 5; the setup consists of the designed EBI probe, an LRC meter (Keysight E4980A, Keysight Technologies Inc., U.S.), material for test, and a laptop for data collection and processing.

For the safety consideration, the electric level of the LCR meter is set to be 10 mV. In the following study, the maximum current at this voltage in all the frequencies is found not to be more than 0.1 mA, which does not cause any tissue damage according to international standards IEC6060-1. In addition, 200 different frequencies from 1 kHz to 2 MHz were applied for each measurement. The frequencies within this range are corresponding to the β-dispersion of biological tissues [40], which can provide rich information about the tissues’ dielectric properties.

### Experimental design

Two experiments were performed in this study, to characterize and evaluate the designed sensor probe.

The first experiment was designed to characterize the electrode sensing capability with saline solutions of different concentrations. Five saline solutions in different concentrations, namely 0.1%, 0.2%, 0.3%, 0.4%, and 0.5% were made and used for the first experiment. During the experiment, the electrode was immersed into the solutions. After waiting for about 5 s, we measured the impedance values 10 times for each saline solution. Then, the collected data were analyzed and compared with the theoretical electrical properties of the saline solutions.

In order to evaluate the overall performance of the designed probe for distinguishing the normal and abnormal states of the same tissue type, the second experiment was designed and conducted with real phantoms based on fresh porcine liver, given that the porcine liver has similar electrical properties as human [12]. Liver tissue is coated with a 40 to 70 μm thick serosal layer [41] which is within the 20% sensitivity range according to Fig. 3. Also, blood or body fluid may contaminate the tissue surface in practice. This effect can be minimized by slightly pressing the probe on the tissue. These factors can be the source of measurement variation and methods to minimize their impact will be a focus in our future study.

To avoid that the EBI data collected on individual liver samples, be affected during each experiment, stabs of cold stored porcine liver was cut into 18 pieces of block with the dimension of 30×30×20 mm. 9 pieces of liver samples remained fresh and the other 9 pieces were soaked in pure water for 5 min to modify the cell architecture of the liver tissue. Normally, the healthy status of tissue can be reflected by its extracellular component and intracellular component. By soaking the liver sample into water, water could be injected into the tissue due to passive transport phenomena [33] and thus change these parameters.

During the second experiment, we first cleaned the liver surface to ensure its dryness. Then the probe was placed on the surface of a liver sample and we slight pressed the probe. According to study [39], the applied force between the probe and the tissue can cause change of electrical property of the tissue. Thus, the experimenter controlled the pressing force based on visual inspection to ensure that the electrodes was contacting the tissue sample well but not causing too much deformation of the tissue. Then the EBI values were measured by the LCR meter. For each sample, the measurements were repeated 10 times, and the data were collected to the laptop for processing. As mentioned in Section *Algorithms for tissue classification*, this study tested and compared the two constructed classifiers, *ℱ_LDA_* and *ℱ_QDA_* modelling the probability of a class *y* given sample *x_i_*. The collected input-output pairs {*x_t_*, *y_t_*} were accumulated as one training dataset ${𝒫}_{t}^{A}$ for establishing the classifiers and the other one as testing dataset ${𝒫}_{t}^{B}$ for evaluating the performance of the classifiers. Here, 25% cross validation was used for verification. The experimental results were compared and presented in Section *Ex vivo tissue results*.

### Ethical approval

The conducted research is not related to either human or animal use.

## Experimental results

### Experimental results of saline solutions

**Fig.6:**
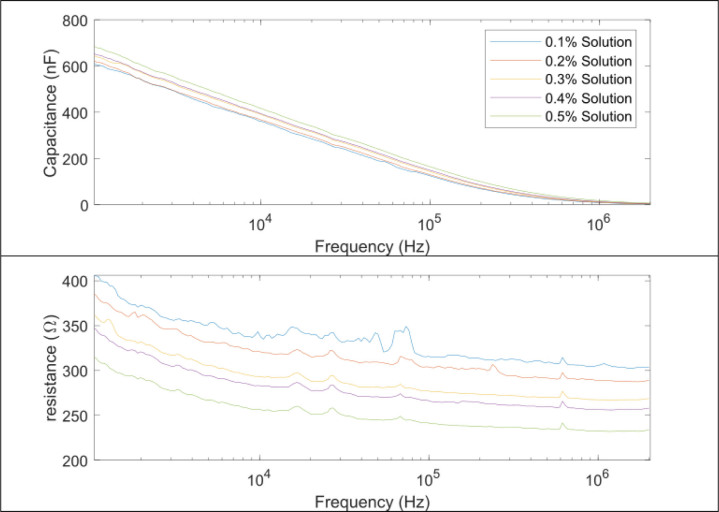
Experimental results of 5 saline solutions in different excitation frequencies with the sensor probe.

Fig. 6 shows the measurement results of different saline solutions using the designed probe. Each curve represents the mean values measured in one saline solution. The *x* axis is the excitation frequency in logarithmic scale and the *y* axis represent the corresponding capacitance values and resistance values, respectively.

The experimental results show that the capacitance values for different saline solutions have little variance (relative standard deviation <4.4%), and this value decreases from 638 nF to 4.4 nF when the excitation frequency increased from 1 kHz to 2 MHz. In contrast, the resistance values were less dependent to the applied frequency. In the low frequency range from 1 kHz to 10 kHz, the resistance values showed a relatively bigger change due to the electrode polarization effect. When higher excitation frequencies (10 kHz to 2 MHz) were applied, the polarization impedance *Z_epi_* became small and contributed less to the resistance value.

### Ex vivo tissue results

**Fig.7:**
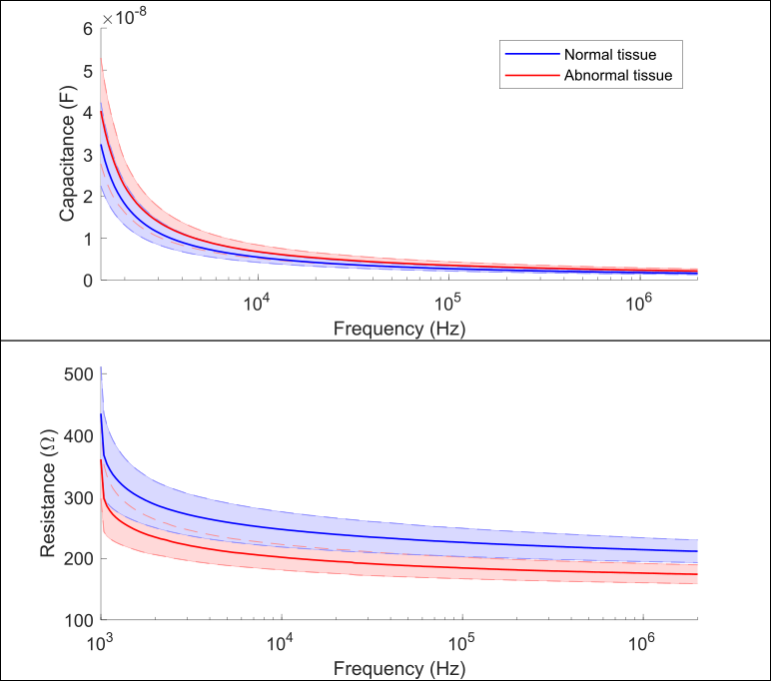
Experimental results of *ex vivo* porcine liver tissues including the capacitance and resistance: the blue line indicates the results of normal tissue and the red line indicates the results of the abnormal tissue.

The capacitance and resistance values of *ex vivo* tissues measured by the designed probe at different excitation frequencies are shown in Fig. 7. Here, we use ‘Normal tissue’ to denote fresh liver samples, and ‘Abnormal tissue’ to denote water soaked tissues. The results of normal tissue samples are plotted in blue lines with a semi-transparent shading representing the standard deviation, while the results of abnormal tissue samples are shown in red.

Then the data analysis was proceeded. First, the Kolmogorov-Smirnov method [34] was used to test whether the collected results are normally distributed. The test results indicate that the p-values of both classes were >0.05. Therefore, the null hypothesis can be rejected for both classes, and the discriminant analysis method is legit for the data classification. In total, 160 data were collected, and 1 data was rejected due to unexpected big noise. Among the collected data samples, 119 data were randomly selected as a training dataset, and 40 data were used as a testing dataset. After the training procedure, the LDA classifier was found to achieve around 100% accuracy and the QDA classifier achieved a 79% accuracy with the training dataset.

Subsequently, the testing dataset were used for evaluating both classifiers. The classification accuracy was 100% for the LDA classifier, and 80% for the QDA classifier. Therefore, a further investigation of the QDA method was carried out and the confusion matrix of the QDA classification results is shown in Tab. 1. According to the classification results, 8 false positive cases were found in the test dataset.

**Tab.1:** Confusion Matrix showing the classification results using the QDA classifier.

	Abnormal	Normal
Abnormal	12	8
Normal	0	20

## Discussion

According to the experimental results of the saline solutions study, the designed sensor probe is found to be able to reflect the electrical property of the testing material effectively. Also, when a higher excitation frequency was applied, the measured capacitance of the saline solutions showed a drastic decline, but relatively less influences were found to the resistance values. This finding was coherent to the International Standard IEC60746-3 which indicates that saline solutions in high frequency measurements can be considered as pure conductive material.

In addition, the *ex vivo* tissue experiments provide a more in-depth evaluation of the sensor probe and the classification methods. The experimental results in Fig.7 reveal the exponential decline tendency for both capacitance and resistance against frequency. This phenomenon is described as the Cole model as mentioned in Section *Modeling of tissue electrical bioimpedance*. Compared to the impedance values of fresh liver tissue, the capacitance of the water-soaked liver samples was slightly higher and their resistance value was found to be lower. This is because the intra-cellular water and extra-cellular water of the liver tissue increased by the overall effect of passive transport and water diffusion. Specifically, the volume of extracellular fluid may increase significantly since a relatively low resistivity in the low frequency range was observed. Since the electrical property of tissue is sensitive to the tissue status change, it implies a great potential of this sensory probe to detect different statuses even for the same tissues.

Also, the experimental results show that both classifier can successfully distinguish normal and abnormal tissue with quite high accuracy (100% for LDA, and 80% for QDA). The LDA classifier has a higher accuracy than that of the QDA classifier, which can be explained due to the variance-covariance are quite different from the two classes. Since the covariance matrix of each class are used in the QDA method, its forming boundary may be affected and result in an overfit. In addition, the collected data set was used to train and test a support vector machine (SVM) based classifier. An accuracy of 98% was found which is higher than the QDA classifier but slightly lower than the LDA classifier. In the future work, more experiments will be performed, and a larger training dataset will be used for training the classifier.

The pressing force on the tissue can be one source of the measurement variations. However, the probe should still be able to classify normal and cancerous liver tissues since their differences in terms of electrical properties are reported to be more significantly [19]. The current version of the probe will require the user to manipulate it based on visual information. Specifically, the user should control the probe to slightly press on the tissue without causing too much deformation of the tissue. This can be difficult in practice. To increase the measurement accuracy, involving a force sensor or a force regulation mechanism into the probe will be investigated in our future work.

## Conclusions

In this study, we propose and develop an EBI technology based sensor probe that can be used for assisting the surgeons in detecting abnormal tissue during a laparoscopic surgery. The probe is designed with an optimized electrode plate to improve the signal-noise ratio and a compact size to be grasped by a forceps for scanning the tissue conveniently. Two tissue classifiers are developed based on supervised learning algorithms. To verify the performance of the designed sensor probe, ex vivo experiments are performed. The experimental results show that the sensor probe can provide effective tissue measurements with quite high accuracy.

The future work will focus on the further experimental studies for the sensor characterization and evaluation, and ex vivo human healthy and pathological tissues will be considered. Furthermore, advanced classification algorithms will be utilized/developed in order to construct a more effective abnormal tissue detector.

## References

[1] Soper NJ, Brunt LM, Kerbl K (1994). Laparoscopic general surgery. New England Journal of Medicine.

[2] Subramonian K, DeSylva S, Bishai P, Thompson P, Muir G (2004). Acquiring surgical skills: a comparative study of open versus laparoscopic surgery. European Urology.

[3] Nagtegaal ID, van de Velde CJ, van der Worp E, Kapiteijn E, Quirke P, van Krieken J (2002). Macroscopic evaluation of rectal cancer resection specimen: Clinical significance of the pathologist in quality control. Journal of Clinical Oncology.

[4] Kaczmarek BF, Sukumar S, Petros F, Trinh QD, Mander N, Chen R, Menon M, Rogers CG (2013). Robotic ultrasound probe for tumor identification in robotic partial nephrectomy: Initial series and outcomes. International Journal of Urology.

[5] Sound S, Okoh AK, Bucak E, Yigitbas H, Dural C, Berber E (2016). Intraoperative tumor localization and tissue distinction during robotic adrenalectomy using indocyanine green fluorescence imaging: a feasibility study. Surgical Endoscopy.

[6] Beccani M, Di Natali C, Sliker LJ, Schoen JA, Rentschler ME, Valdastri P (2013). Wireless tissue palpation for intraoperative detection of lumps in the soft tissue. IEEE Transactions on Biomedical Engineering.

[7] Escoto A, Bhattad S, Shamsil A, Sanches A, Trejos AL, Naish MD, Malthaner RA, Patel RV A multi-sensory mechatronic device for localizing tumors in minimally invasive interventions.

[8] Gafford JB, Kesner SB, Degirmenci A, Wood RJ, Howe RD, Walsh CJ A monolithic approach to fabricating low-cost, millimeter-scale multi-axis force sensors for minimally-invasive surgery.

[9] McKinley S, Garg A, Sen S, Kapadia R, Murali A, Nichols K, Lim S, Patil S, Abbeel P, Okamura AM, Goldberg K A single-use haptic palpation probe for locating subcutaneous blood vessels in robot-assisted minimally invasive surgery.

[10] Ahn B, Kim Y, Oh CK, Kim J (2012). Robotic palpation and mechanical property characterization for abnormal tissue localization. Medical & Biological Engineering & Computing.

[11] Guo J, Xiao B, Ren H (2019). Compensating Uncertainties in Force Sensing for Robotic-Assisted Palpation. Applied Sciences.

[12] Carrara N An Internet resource for the calculation of the ‘Dielectric Properties of Body Tissues’ in the frequency range 10 Hz-100 GHz.

[13] Khalil SF, Mohktar MS, Ibrahim F (2014). The theory and fundamentals of bioimpedance analysis in clinical status monitoring and diagnosis of diseases. Sensors.

[14] Cheng Z, Davies BL, Caldwell DG, Mattos LS (2018). A new venous entry detection method based on electrical bio-impedance sensing. Annals of Biomedical Engineering.

[15] Naranjo-Hernández D, Reina-Tosina J, Min M (2019). Fundamentals, recent advances, and future challenges in bioimpedance devices for healthcare applications. Journal of Sensors.

[16] Yu D, Jun D, Qing Y, Jianxun Z (2015). Development of a noninvasive electrical impedance probe for minimally invasive tumor localization. Physiological Measurement.

[17] Emran S, Lappalainen R, Kullaa AM, Myllymaa S (2018). Concentric ring probe for bioimpedance spectroscopic measurements: design and ex vivo feasibility testing on pork oral tissues. Sensors.

[18] Cheng Z, Carobbio AL, Soggiu L, Migliorini M, Guastini L, Mora F, Fragale M, Ascoli A, Africano S, Caldwell D, Canevari FR (2020). SmartProbe: a bioimpedance sensing system for head and neck cancer tissue detection. Physiological Measurement.

[19] Laufer S, Ivorra A, Reuter VE, Rubinsky B, Solomon SB (2010). Electrical impedance characterization of normal and cancerous human hepatic tissue. Physiological Measurement.

[20] Yang L, Liu W, Chen R, Zhang G, Li W, Fu F, Dong X (2017). In vivo bioimpedance spectroscopy characterization of healthy, hemorrhagic and ischemic rabbit brain within 10 Hz-1 MHz. Sensors.

[21] Ollmar S, Grant S (2016). Nevisense: improving the accuracy of diagnosing melanoma. Melanoma management.

[22] O'rourke AP, Lazebnik M, Bertram JM, Converse MC, Hagness SC, Webster JG, Mahvi DM (2007). Dielectric properties of human normal, malignant and cirrhotic liver tissue: in vivo and ex vivo measurements from 0.5 to 20 GHz using a precision open-ended coaxial probe. Physics in Medicine & Biology.

[23] Halter RJ, Kim YJ (2014). Toward microendoscopic electrical impedance tomography for intraoperative surgical margin assessment. IEEE Transactions on Biomedical Engineering.

[24] Lee BR, Roberts WW, Smith DG, Ko HW, Epstein JI, Lecksell K, Partin AW (1999). Bioimpedance: novel use of a minimally invasive technique for cancer localization in the intact prostate. The Prostate.

[25] Cheng Z, Davies BL, Caldwell DG, Mattos LS A venipuncture detection system for robot-assisted intravenous catheterization.

[26] Dai Y, Du J, Yang Q, Zhang J (2014). Noninvasive electrical impedance sensor for in vivo tissue discrimination at radio frequencies. Bioelectromagnetics.

[27] Cheng Z, Dall'Alba D, Foti S, Mariani A, Chupin TJ, Caldwell DG, Ferrigno G, De Momi E, Mattos LS, Fiorini P (2019). Design and integration of electrical bio-impedance sensing in surgical robotic tools for tissue identification and display. Frontiers in Robotics and AI.

[28] Cole KS, Cole RH (1941). Dispersion and absorption in dielectrics I. Alternating current characteristics. The Journal of chemical physics.

[29] Trebbels D, Fellhauer F, Jugl M, Haimerl G, Min M, Zengerle R (2011). Online tissue discrimination for transcutaneous needle guidance applications using broadband impedance spectroscopy. IEEE Transactions on Biomedical Engineering.

[30] Grimnes S, Martinsen OG (2008). Bioimpedance and bioelectricity basics.

[31] Štulík K, Amatore C, Holub K, Mareček V, Kutner WŁ (2000). Microelectrodes. Definitions, characterization, and applications. Pure Appl. Chem.

[32] Cheng Z, Dall'Alba D, Caldwell DG, Fiorini P, Mattos LS Design and Integration of Electrical Bio-Impedance Sensing in a Bipolar Forceps for Soft Tissue Identification: A Feasibility Study.

[33] Gu W, Lai W (1998). A mixture theory for charged-hydrated soft tissues containing multi-electrolytes: Passive transport and swelling behaviors. Journal of Biomechanical Engineering.

[34] Simard R, L'Ecuyer P (2011). Computing the two-sided Kolmogorov-Smirnov distribution. Journal of Statistical Software.

[35] Salvador B, Franco E, Perdigones F, Quero JM (2017). Fabrication process for inexpensive, biocompatible and transparent PCBs. Application to a flow meter. Microelectronic Engineering.

[36] Fournier-Desseux A, Jossinet J (2005). Assessment of 1-lead and 2-lead electrode patterns in electrical impedance endotomography. Physiological Measurement.

[37] Keshtkar A, Salehnia Z, Somi MH, Eftekharsadat AT (2012). Some early results related to electrical impedance of normal and abnormal gastric tissue. Physica Medica.

[38] Mirtaheri P, Grimnes S, Martinsen ØG (2005). Electrode polarization impedance in weak NaCl aqueous solutions. IEEE Transactions on Biomedical Engineering.

[39] Ruiz-Vargas A, Ivorra A, Arkwright JW (2018). Design, construction and validation of an electrical impedance probe with contact force and temperature sensors suitable for in-vivo measurements. Scientific reports.

[40] Gabriel S, Lau RW, Gabriel C (1996). The dielectric properties of biological tissues: III. Parametric models for the dielectric spectrum of tissues. Physics in Medicine & Biology.

[41] MacSween RN, Anthony PP, Scheuer PJ (1979). Pathology of the liver.

